# Synergism between Medihoney and Rifampicin against Methicillin-Resistant *Staphylococcus aureus* (MRSA)

**DOI:** 10.1371/journal.pone.0057679

**Published:** 2013-02-28

**Authors:** Patrick Müller, Dagmar G. Alber, Lynne Turnbull, Ralf C. Schlothauer, Dee A. Carter, Cynthia B. Whitchurch, Elizabeth J. Harry

**Affiliations:** 1 The ithree institute, University of Technology Sydney (UTS), Sydney, New South Wales, Australia; 2 Comvita NZ Limited, Te Puke, New Zealand; 3 School of Molecular Bioscience, University of Sydney, Sydney, New South Wales, Australia; Rockefeller University, United States of America

## Abstract

Skin and chronic wound infections caused by highly antibiotic resistant bacteria such as methicillin-resistant *Staphylococcus aureus* (MRSA) are an increasing and urgent health problem worldwide, particularly with sharp increases in obesity and diabetes. New Zealand manuka honey has potent broad-spectrum antimicrobial activity, has been shown to inhibit the growth of MRSA strains, and bacteria resistant to this honey have not been obtainable in the laboratory. Combinational treatment of chronic wounds with manuka honey and common antibiotics may offer a wide range of advantages including synergistic enhancement of the antibacterial activity, reduction of the effective dose of the antibiotic, and reduction of the risk of antibiotic resistance. The aim of this study was to investigate the effect of Medihoney in combination with the widely used antibiotic rifampicin on *S. aureus*. Using checkerboard microdilution assays, time-kill curve experiments and agar diffusion assays, we show a synergism between Medihoney and rifampicin against MRSA and clinical isolates of *S. aureus*. Furthermore, the Medihoney/rifampicin combination stopped the appearance of rifampicin-resistant *S. aureus in vitro*. Methylglyoxal (MGO), believed to be the major antibacterial compound in manuka honey, did not act synergistically with rifampicin and is therefore not the sole factor responsible for the synergistic effect of manuka honey with rifampicin. Our findings support the idea that a combination of honey and antibiotics may be an effective new antimicrobial therapy for chronic wound infections.

## Introduction

Infectious diseases continue to take a toll on human health and life expectancy. In the western world, increased longevity and health complications due to the sharp rise in obesity and diabetes have made chronic wound infections particularly problematic. In the United States, chronic wounds affect 6.5 million patients and are estimated to cost US$25 billion annually, with significant increases expected in the future [1]. Treatment of these infections is becoming increasingly difficult due to antibiotic resistance to currently available drugs [2]. *Staphylococcus aureus* is the causative agent of many serious acute and chronic skin infections and is one of the most predominant wound pathogens [3], [4], [5]. Strains of methicillin-resistant *S. aureus* (MRSA) have become increasingly common and the spread of these represents a serious health threat [6]. Commercial development of new classes of antibiotics has diminished over the past 15 years and few pharmaceutical companies remain active in this area [7]. There is an urgent need for new approaches to treat these infections.

To combat antibiotic resistance, combination antibiotic treatment is widely practiced in the clinic. Such treatment can result in synergism to provide increased efficacy and a reduction in amount of each antibiotic used, which can reduce the risk of possible side effects and treatment costs [8], [9], [10], [11]. Moreover, combination use of antibiotics with different modes of action reduce the risk of antibiotic resistance arising during therapy [12], [13]. This is particularly important for chronic wounds where antibiotic therapy is often long-term.

Given the difficulty in treating infected chronic wounds due to multi-resistant bacteria, honey is increasingly being used as a topical treatment for these wounds. There are several reports of its successful application in the treatment of chronic wound infections not responding to antibiotic therapy [14]. The major honey in medical use today, manuka honey, is available in various licensed dressings and is sourced from the New Zealand manuka tree *Leptospermum scoparium*. Manuka honey has broad-spectrum antibacterial activity [15], [16], [17], [18] and is effective against antibiotic-resistant wound pathogens [17], [19], [20]. Furthermore, no resistant bacteria could be isolated after exposure of wound isolates (*Escherichia coli*, MRSA, *Pseudomonas aeruginosa,* and *Staphylococcus epidermidis*) to sub-inhibitory concentrations of manuka honey [20], [21]. This is believed to be due to the fact that manuka honey contains a range of antibacterial constituents including methylglyoxyl (MGO) [22], [23]; hydrogen peroxide [24], [25], [26], and other active substances that are yet to be defined [26].

The broad-spectrum antibiotic rifampicin is commonly used in the treatment of staphylococcal prosthesis- or skin-associated infections, including chronic wounds [27], [28]. The chemical structure of rifampicin allows this drug to penetrate well into tissues and abscesses, which are poorly penetrated by most other anti-staphylococcal agents [29], [30]. However, *S. aureus* can develop rifampicin resistance during a single passage [29], and it is therefore always used in combination with other antibiotics to treat bacterial infections [30], [31], [32], [33]. The development of resistance to rifampicin in bacteria is typically due to a single, but variable, point mutation in its target, the β subunit of bacterial RNA polymerase [34], [35], [36]. Although rifampicin combination therapy has been demonstrated to be effective against severe staphylococcal infections, rifampicin resistance can still emerge [37].

A combination of the antimicrobial properties of clinically approved antibiotics and the antibacterial activity of manuka honey could lead to a new spectrum of antimicrobials that have the potential to prevent the emergence of resistant bacterial strains, providing broad-spectrum coverage and consequently improving therapeutic efficiency. In this study, we show a synergistic effect between rifampicin and commercially available FDA-approved manuka honey, Medihoney (Comvita, NZ) on clinical *S. aureus* isolates, including MRSA strains. Unlike with rifampicin alone, in which resistance was observed after overnight incubation on plates, the combination of Medihoney and rifampicin maintained susceptibility of *S. aureus* to rifampicin. We also show that MGO is not solely responsible for the observed synergistic action between rifampicin and Medihoney. This study highlights the potential of a combinational use of Medihoney and rifampicin to develop novel therapies for chronic wounds and serious skin infections, to both improve efficacy and reduce the risk of antibiotic resistance.

## Materials and Methods

### *S. aureus* strains, Media and Antibiotics

Laboratory strain *S. aureus* NCTC8325 and several *S. aureus* clinical isolates were used in this study. The latter included non-MRSA strains, 04-229-2455 and 04-227-3567 and MRSA strains, IMVS67 (nmMRSA D), MW2 (USA400, CA-MRSA), and RPAH18 (Aus-2) (kindly provided by Dr. Jon Iredell, Westmead Hospital, Sydney) and USA300 (CA-MRSA) (kindly provided by Dr. Barry Kreiswirth, Public Health Research Institute Center, Newark, NJ). All growth assays were set up in cation-adjusted Mueller Hinton II Broth (CaMHB, Becton Dickinson). Rifampicin, oxacillin and methyglyoxal (MGO; 40% w/v in water) were obtained from Sigma-Aldrich.

### Honey

Two types of honeys were used in this study: commercially available active manuka honey in a proprietary formulation (Medihoney, Comvita Ltd, NZ) [20], [38], [39] and manuka honey sourced from *Leptospermum scoparium* plantations in Hokianga, NZ (provided by Comvita Ltd, NZ). Honey concentrations are reported here as % weight/volume. MGO levels were determined during the study to be 958 mg/kg for manuka honey and 781 mg/kg for Medihoney (Comvita Ltd, NZ) [40]. A sugar solution containing 7.5 g sucrose, 37.5 g maltose, 167.5 g glucose, 202.5 g fructose (all from Sigma-Aldrich) in 85 mL sterile deionised water was used to mimic the sugar content of honey.

### Determination of Minimal Inhibitory Concentration (MIC) in Microtiter Plates

MGO and honey were diluted in CaMHB. Honey concentrations, varying by 1% (range 1–32%), were used in successive wells. Microtiter plates were then inoculated with approximately 10^7 ^CFU/mL (determined by CFU counting) of *S. aureus*. The MIC of rifampicin was determined by serial doubling dilution with DMSO. Final concentrations of 2% DMSO in CaMHB were used in the experiments. Controls included a serial dilution of lincomycin (to assess plate-to-plate variation), a positive control with bacteria alone in CaMHB (with 2% DMSO for rifampicin) and a negative control (no bacteria) with CaMHB (containing 2% DMSO for rifampicin). Plates were incubated at 37°C for 22 h and the 595 nm was measured using a Synergy HT Bio-Tek plate reader. The MICs were defined as the lowest concentration of rifampicin, MGO, and honey (alone or in combination) that inhibited growth by 99.9% compared to the no-treatment control.

### Checkerboard Microdilution Assay

Rifampicin was serially diluted in DMSO and each dilution was added, in duplicate, to a 96-well plate to a final DMSO concentration of 2%. MGO was diluted in CaMHB. Prior to the addition of bacteria to the wells, a 50% honey solution in CaMHB was prepared, and serial dilutions were made. Then, an overnight culture of *S. aureus* NCTC8325 was diluted and approximately 10^7^ CFU/mL were added to each well. Plates were handled as described above. Each experiment was performed in duplicate three times on different days.

The fractional inhibitory concentration index (FICI) was calculated as the sum of the MIC of each compound when used in combination divided by the MIC of the compound used alone. Synergy and antagonism were defined by an FICI of ≤0.5 and >4, respectively. An FIC index of >0.5 but ≤4 was considered indifferent [41], [42].

### Agar Diffusion Test

Fifty µL aliquots of 10^9^ CFU/mL overnight culture of each of the *S. aureus* strains were spread uniformly onto tryptic soy broth agar (TSA, Oxoid) with or without 5% honey (or sugar solution) in 60×15 mm tissue culture plates (Falcon). Paper discs impregnated with 4 µg of each antibiotic were then placed onto the agar surface. Inhibition zones were measured after incubation at 37°C for 24 h. Assays were performed three times in duplicate. In order to determine the effect of honey alone, bacterial CFUs were determined by a standard plate count method as follows. Twenty µL of overnight culture (approximately 1×10^9^ CFU/mL) were diluted in 180 µL of PBS, followed by further serial dilution (10^−1^ to 10^−8^). Twenty µL of each dilution was then spotted onto a freshly prepared TSA plate with or without 5% honey (in triplicate). Colonies were counted after incubation at 37°C for 24 h, and CFUs determined.

### Time-kill Curves

An exponentially growing culture of *S. aureus* NCTC8325 was diluted to 1×10^7^ CFU/mL in CaMHB for inoculation. The test concentrations were 0.2 µg/mL rifampicin, 7% Medihoney, 70 µg/mL MGO, 70 µg/mL MGO in a sugar solution corresponding to 7% honey (MGO^S^), a combination of 0.2 µg/mL of rifampicin and 7% Medihoney, a combination of 0.2 µg/ml of rifampicin and 70 µg/ml MGO, and a combination of 0.2 µg/ml of rifampicin and 70 µg/ml MGO^S^. At pre-determined time points (0, 2, 4, 8, 12, 24 and 48 h after incubation with agitation at 37°C) a 20 µL aliquot was removed from each culture and serially diluted 10-fold in CaMHB. The dilutions were used for CFU counting as described above except TSA plates without honey were used. Synergism and antagonism were defined as either an increase or decrease, respectively, of ≥2 log_10_-CFU/mL in antibacterial activity produced by the combination compared to that by the more active agent alone after 24 h, while a change of <2 log10 CFU/mL was considered indifferent [43]. All CFU counting was done in duplicate. All statistical analyses were performed with GraphPad Prism Statistical Software 6.0 (GraphPad Software, Inc. La Jolla, Ca).

## Results

### Synergistic Activity between Medihoney and Rifampicin

The antimicrobial activity of Medihoney and manuka honey was confirmed by determining the minimum inhibitory concentration (MIC) against *S. aureus* NCTC8325. Both honeys gave an MIC of 8% (w/v). The MIC of rifampicin was 0.039 µg/mL (**Table S1**). The MICs of rifampicin and Medihoney for the clinical isolates (including MRSA strains) were similar, ranging from 6–8% honey and 0.039–0.078 µg/mL rifampicin (**Table S1**) and are comparable to MICs reported in the literature [44], [45], [46].

To test whether there was any synergy between rifampicin and Medihoney on *S. aureus* a checkerboard microdilution assay was performed. The results of the checkerboard analysis are summarized in **Table 1**. An increased sensitivity against rifampicin was observed in combination with Medihoney against the laboratory *S. aureus* strain NCTC8325 and both MRSA (RPAH18, IMVS67 and MW2) and non-MRSA (04-227-3567) clinical isolates. The corresponding FICIs were ≤0.5 in all tested strains (**Table 1**), demonstrating a synergistic effect [41], [42]. A synergistic effect was also seen with manuka honey and rifampicin (FIC ≤0.5; **Table 1**). However, rifampicin in combination with the sugar solution was not synergistic (data not shown).

**Table 1:** Interaction of Medihoney and rifampicin against *S. aureus* by checkerboard microdilution assay.

			MIC_Rif_ a (µg/ml)		MIC_honey_ b (%[w/v])		FICI	synergistic
			alone	comb.	alone	comb.		
NCTC8325	Rif	Medihoney	0.039	0.0024	8	3	0.45 (0.07+0.38)	yes
	Rif	manuka	0.039	0.0024	8	3	0.45 (0.07+0.38)	yes
RPAH 18^1^	Rif	Medihoney	0.078	0.0024	8	3	0.41 (0.03+0.38)	yes
	Rif	manuka	0.078	0.0024	8	3	0.42 (0.03+0.38)	yes
MW2^1^	Rif	Medihoney	0.039	0.0024	8	3	0.45 (0.07+0.38)	yes
	Rif	manuka	0.039	0.0024	8	3	0.45 (0.07+0.38)	yes
IMVS67^1^	Rif	Medihoney	0.078	0.0024	8	3	0.41 (0.03+0.38)	yes
	Rif	manuka	0.078	0.0024	8	3	0.41 (0.03+0.38)	yes
04-227-3567^2^	Rif	Medihoney	0.039	0.0024	8	3	0.45 (0.07+0.38)	yes
	Rif	manuka	0.039	0.0024	8	3	0.45 (0.07+0.38)	yes

1 MRSA strain;

2 clinical isolate;

a MIC_Rif_ is minimum inhibitory concentration of rifampicin either alone (alone) or in combination with honey (comb.);

b MIC_honey_ is the MIC of honey (Medihoney and manuka honey, respectively) either alone or in combination with rifampicin; Rif is rifampicin.

**Figure 1:**
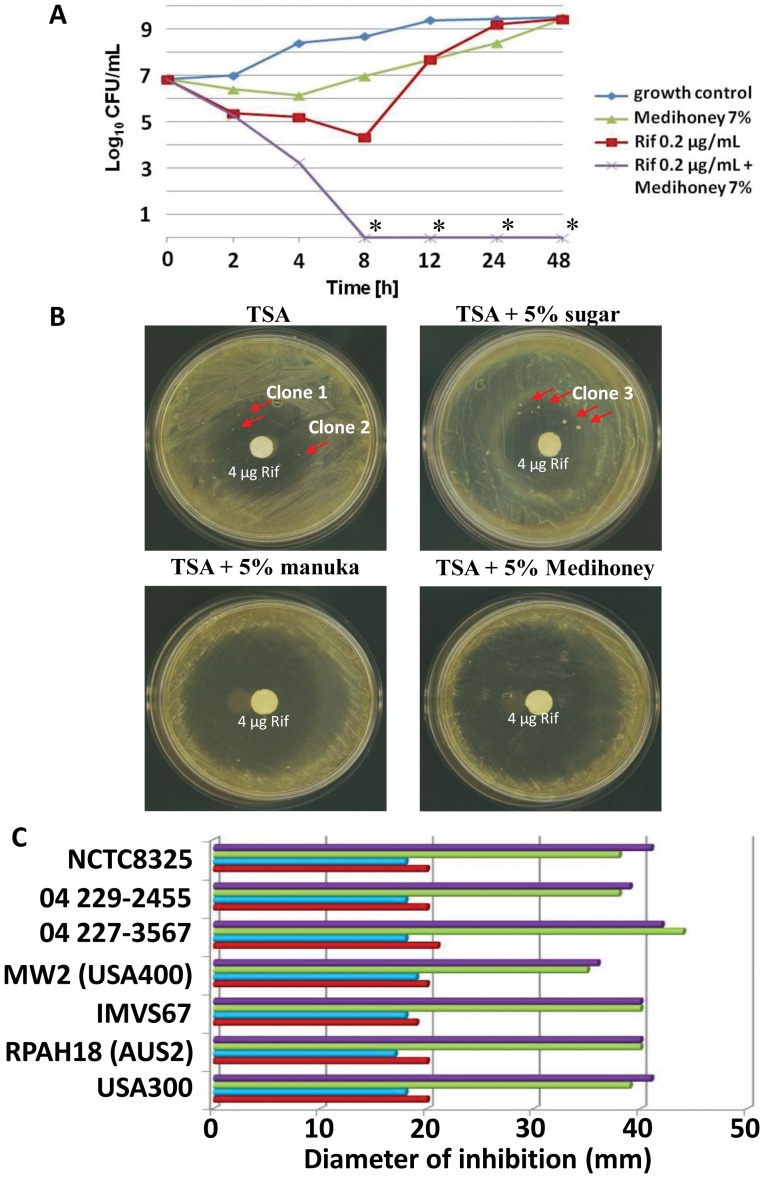
Enhanced antibacterial activity of rifampicin-honey combination treatment against *S. aureus*. (**A**) Time-kill curves for *S. aureus* NCTC8325 in CaMHB. Bacteria were incubated in 7% Medihoney, 0.2 µg/ml rifampicin, or both. A growth control using just CaMHB is included as indicated. Rif: rifampicin; *: below detection limit (<50 CFU/ml). (**B**) Filter discs containing 4 µg of rifampicin were placed on *S. aureus* NCTC8325 spread on TSA plates containing no honey (TSA), 5% sugar solution (sugar), 5% manuka honey, or 5% Medihoney. The shown plates were incubated at 37°C for 24 h. Red arrows denote rifampicin resistant colonies that appeared on the TSA and sugar control plates, but not on Medihoney or manuka honey plates. Minimum inhibitory concentrations of break-through colonies (clones 1–3) were determined against Medihoney and rifampicin (see Fig. 4). (**C**) Sensitivity of different *S. aureus* strains to rifampicin and honey using the agar disc diffusion assay. Diameter (in mm) of zones of inhibition around 4 µg-impregnated rifampicin discs on TSA plates without honey (red bars), and in the presence of either 5% sugar solution (blue bars), 5% manuka honey (green bars) or 5% Medihoney (black bars).

To confirm the synergistic activity between rifampicin and Medihoney, time-kill experiments were performed (**Fig. 1A**). With an initial inoculum of 10^7^ CFU/mL, 7% Medihoney alone (sub-MIC level) slowed down bacterial growth. However, growth of bacteria then increased and by 24 h, bacterial growth in the presence of 7% Medihoney was at the same level as no treatment. Rifampicin alone also completely inhibited bacterial growth up to 8 h of incubation. However, even at 0.2 µg/mL (∼5×MIC) the CFU/mL count increased dramatically after 8 h to levels of growth similar to that observed in the untreated cultures at 24 h. This is due to the attainment of resistance to this antibiotic by *S. aureus* (see below). A combination of 7% Medihoney and 0.2 µg/mL rifampicin yielded a >2-log_10_ decrease in CFU/mL compared to rifampicin or Medihoney alone, and this was sustained up to 48 h (**Fig. 1A**). This is considered to be a synergistic activity [43]. Similar results were observed with rifampicin plus manuka honey at the same concentrations (data not shown).

Agar disc diffusion tests were performed to visualize the synergistic interaction between rifampicin and Medihoney with *S. aureus* (**Fig. 1B**). The mean diameter of the inhibitory zone for 4 µg rifampicin on a filter disc was 20 mm on TSA plates and 18 mm on TSA plates with 5% sugar solution. This zone of inhibition increased markedly to 41 mm and 38 mm on TSA plates containing 5% Medihoney and 5% manuka honey, respectively. All clinical isolates of *S. aureus* tested, including the MRSA strains, gave similar results (**Fig. 1C**). To test whether honey alone was responsible for this effect, we determined the CFU/mL of NCTC8325 grown overnight on TSA plates containing 5% Medihoney or 5% manuka honey. The CFU/mL were only slightly decreased on these plates compared to TSA plates without honey or with 5% sugar solution (**Table 2**), demonstrating that 5% Medihoney alone had no significant effect on the growth of the bacterium on the plates (p>0.05). This result also supports the synergistic antibacterial activity of Medihoney and rifampicin in combination.

**Table 2:** Effect of sub-inhibitory concentration of honey on the growth of *S. aureus* NCTC8325 on agar plates.

	CFU/mL (×10^7^)	% control
TSA	400	1 00
TSA +5% sugar solution	1500	375
TSA +5% manuka honey	350	88
TSA +5% Medihoney	350	88

### MGO is not Solely Responsible for Honey-rifampicin Synergy

MGO is one of the predominant antimicrobial compounds in manuka honey [22], [23]. To investigate whether MGO responsible for the synergistic effect in combination with rifampicin, a checkerboard microdilution assay was performed (**Table 3**). MGO showed antibacterial activity against all tested *S. aureus* strains, with MICs of 150–160 µg/mL. This is comparable to MIC data reported in the literature [22], [23]. These concentrations correspond to the MGO concentration in 16–17% (w/v) of our tested manuka honey (given that manuka honey contains 958 mg/kg MGO). While this might seem high compared to the MIC of an antibiotic, this ubiquitous compound, while toxic, is also beneficial to bacterial cells [47]. Therefore, unlike antibiotics, it is unclear how much MGO is actually harmful and the MIC for MGO may not directly translate like antibiotics. The combination of MGO and rifampicin was not synergistic toward any of the tested *S. aureus* strains (FICI >0.5). In the presence of sugar, at the same concentrations present in the honey experiments, the FICIs were higher (>1), indicating that the combined effect of MGO and rifampicin is weaker in the presence of sugar (**Table 3**).

**Table 3:** Interaction of methylglyoxal and rifampicin against *S. aureus* by checkerboard microdilution assays.

			MIC_Rif_ (µg/ml)		MIC_MGO/MGO_^s^ (µg/ml)		FICI	synergistic	
			alone	comb.	alone	comb.			
NCTC8325	Rif	Medihoney	0.039	0.0039	150	80	0.63 (0.1+0.53)	no	
	Rif	manuka	0.039	0.024	170	150	1.5 (0.62+0.88)		no
RPAH 18^1^	Rif	Medihoney	0.078	0.0078	160	80	0.6 (0.1+0.5)		no
	Rif	manuka	0.078	0.024	170	160	1.25 (0.31+0.94)		no
04-227-3567^2^	Rif	Medihoney	0.039	0.0039	150	80	0.63 (0.1+0.53)		no
	Rif	manuka	0.039	0.0024	160	140	1.5 (0.62+0.88)		no

1 MRSA strain;

2 clinical isolate; MIC is minimum inhibitory concentration; MGO: methylglyoxal in CaMHB; MGO^S^: methylglyoxal in CaMHB with sugar solution (equivalent to the sugar content of honey); Rif is rifampicin.

**Figure 2:**
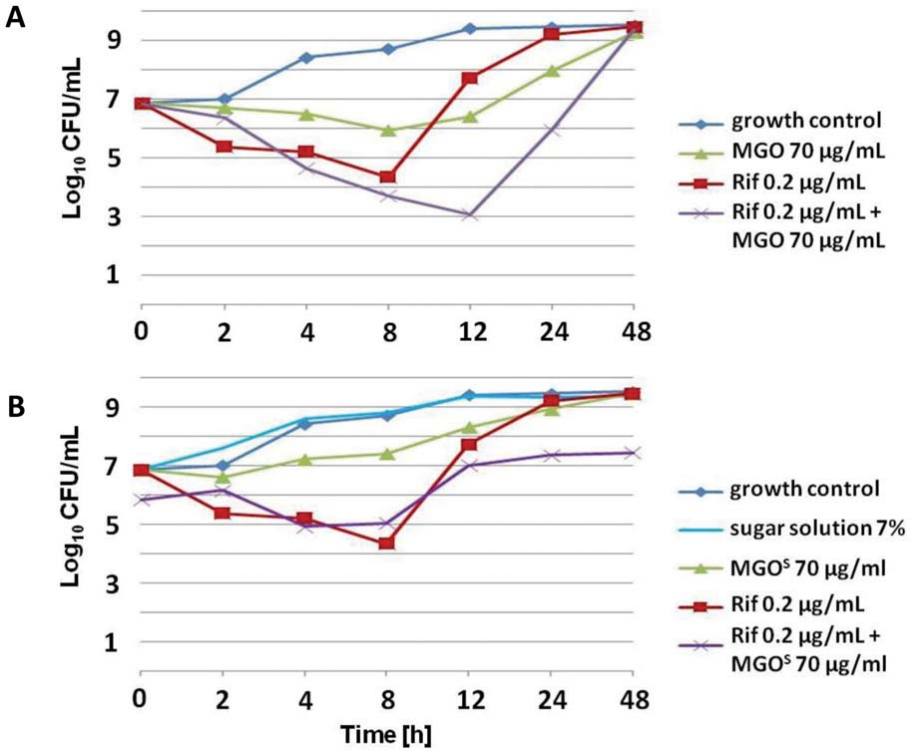
Growth curves of *S. aureus* NCTC8325 in CaMHB. Bacteria were incubated with (**A**) 70 µg/ml MGO, 0.2 µg/ml rifampicin, or both, or with (**B**) 70 µg/ml MGO (in CaMHB with 7% sugar solution, MGO^S^), 0.2 µg/ml rifampicin, or both. A growth control using just CaMHB is included as indicated. Rif is rifampicin.

The synergistic effect of MGO and rifampicin was also examined using time-kill assays. A concentration of 70 µg/ml MGO (corresponding to the concentration of MGO in 7% (w/v) manuka honey) inhibited growth of *S. aureus* NCTC8325 for up to 8 h. However, after 8 h, growth of bacteria occurred and at 48 h, the CFU/mL count increased to levels of growth similar to that observed in the untreated culture **(** **Fig. 2A**). When combined with rifampicin, an increase in the antimicrobial activity could be detected, but after 12 h the CFU/mL count reached the level of the no-treatment control. *S. aureus* isolates originating from that sample and subsequently cultured in the presence of rifampicin were no longer susceptible to rifampicin at all tested concentrations (0–20 µg/ml) (data not shown). MGO in CaMHB medium supplemented with sugar equivalent to that present in 7% honey (MGO^S^) had reduced antimicrobial activity compared to MGO in CaMHB (**Fig. 2B**).

These results demonstrate that, although MGO alone displays a clear antibacterial activity, MGO is not the sole reason for the antimicrobial activity of manuka honey. More importantly, while a combinational treatment of MGO and rifampicin resulted in increased sensitivity of *S. aureus* to rifampicin, unlike honey this effect was only additive, not synergistic, and did not result in complete inhibition of growth.

### No Reversal of Rifampicin Resistance after Treatment with Medihoney

**Figure 3:**
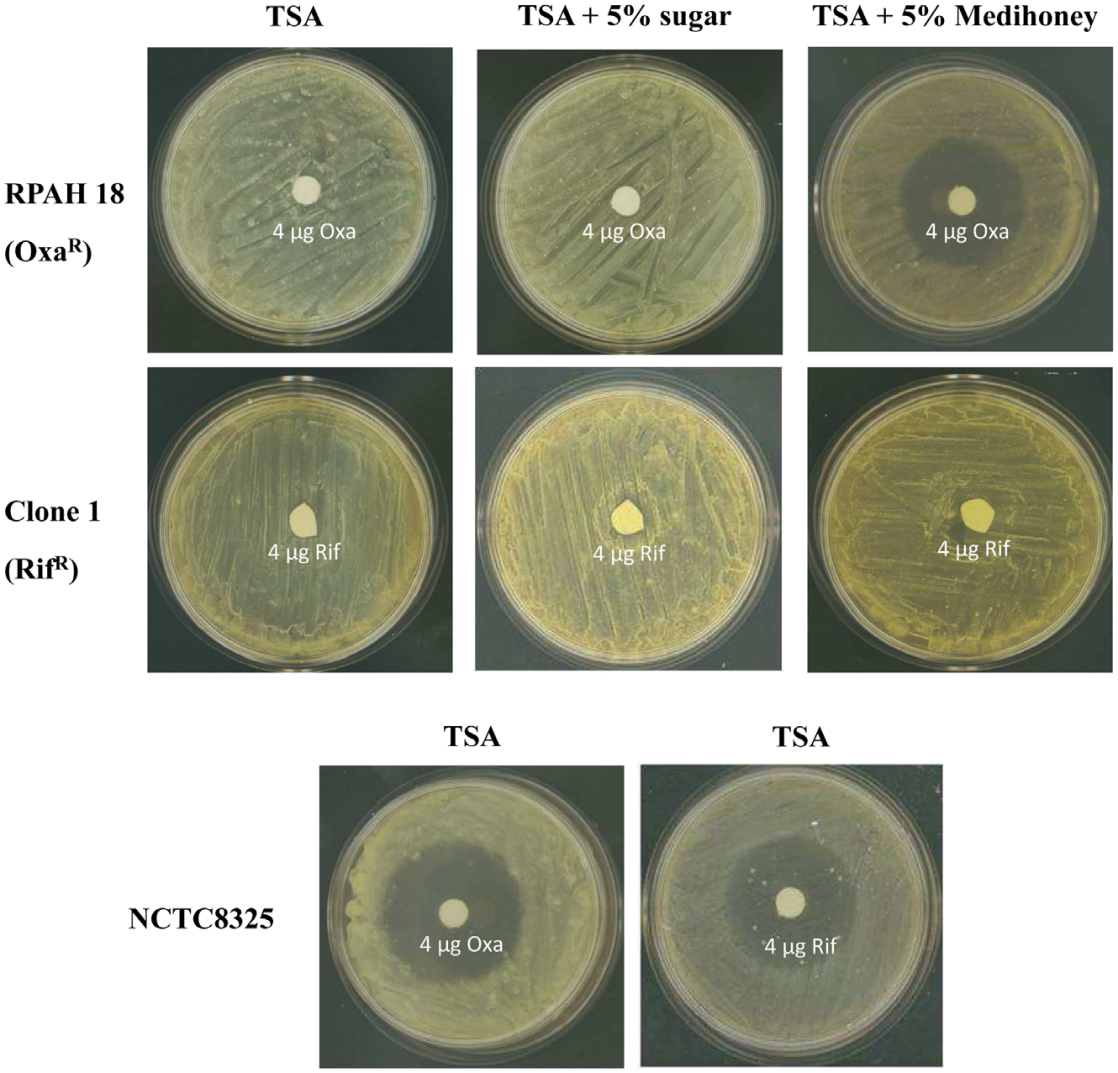
Reversal of oxacillin resistance but not rifampicin resistance in *S. aureus* by Medihoney. Oxacillin resistant MRSA RPAH18 and rifampicin resistant clone 1 (Fig. 1) were streaked out on TSA plates containing no honey (TSA), 5% sugar solution (sugar), or 5% Medihoney. Inhibition zones around filter discs containing 4 µg rifampicin (rif) or 4 µg oxacillin (oxa) were measured after incubation at 37°C for 24 h.

It has been reported, that a combination of oxacillin and manuka honey can restore oxacillin susceptibility to MRSA strains [44]. In order to investigate, whether a combination of rifampicin and Medihoney can reverse rifampicin resistance, an agar disc diffusion assay was performed. The oxacillin resistant strain RPAH18 and a rifampicin resistant NCTC8325 clone (clone 1, refer to **Fig. 1**) were spread out on TSA plates or TSA plates containing 5% Medihoney. Sub-inhibitory concentrations of Medihoney caused the appearance of inhibition zones of 25 mm diameter around 4 µg oxacillin discs, showing the reversal of oxacillin resistance in presence of Medihoney. In contrast, no inhibition zones could be detected around 4 µg rifampicin discs on Medihoney plates (**Fig. 3**). Thus, unlike oxacillin, Medihoney is not able to restore rifampicin susceptibility to *S.aureus* that are already resistant to rifampicin.

### Presence of Medihoney Prevents the Emergence of Rifampicin-resistant *S. aureus*

The results of the time-kill experiments with *S. aureus* NCTC8325 (**Fig. 1A**) showed that 0.2 µg/mL rifampicin displayed antimicrobial activity. However, at 24 h the bacterial CFU/mL was similar to levels of growth observed in the no-treatment cultures. This strongly suggests that the bacteria had developed the ability to grow in the presence of rifampicin. To verify this, we tested *S. aureus* NCTC8325 originating from the rifampicin treated sample (after 24 h) for susceptibility to rifampicin by re-assessing the MIC (examined in the range of 0.0012 to 20 µg/mL rifampicin). In all experiments these bacteria were able to grow at the highest levels of rifampicin when this compound was added alone (data not shown). *S. aureus* NCTC8325 cells originating from the sample with 7% Medihoney were still susceptible to either rifampicin or Medihoney after 24 h (data not shown). However, treatment of previously naive cultures of *S. aureus* NCTC8325 with 0.2 µg/mL rifampicin in combination with 7% Medihoney resulted in a complete inhibition of growth (**Fig. 1A**). These findings suggest that the combination of rifampicin and Medihoney can maintain the susceptibility of *S. aureus* to rifampicin, even at sub-MIC levels of Medihoney.

**Figure 4:**
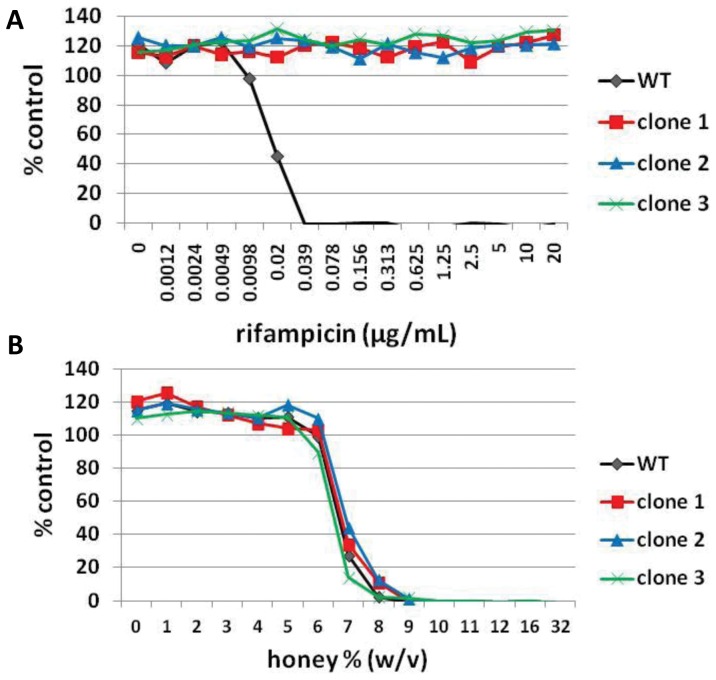
Susceptibility of break-through colonies to rifampicin and Medihoney. Break-through colonies growing in the zone of inhibition around the rifampicin disc on TSA plates (clone 1 and clone 2) and TSA plates with 5% sugar solution (clone 3) were selected (see Fig. 2A). Overnight cultures were prepared in CaMHB and minimum inhibitory concentrations (MIC) of rifampicin (**A**) and Medihoney (**B**) were determined. WT is wild type *S. aureus* NCTC8325.

In the agar disc diffusion assay we observed single break-through colonies within the zone of inhibition on rifampicin-TSA plates and on rifampicin-TSA plates with 5% sugar solution (**Fig. 1A**). Several of these colonies were isolated and tested for susceptibility to rifampicin and honey by determining MICs as described above. All tested clones now had a rifampicin MIC >20 µg/mL but were still fully sensitive to Medihoney (**Fig. 4A and 4B**). No colonies could be detected in the zone of clearance on rifampicin-TSA plates containing 5% Medihoney or manuka honey, even after 48 h incubation (data not shown), indicating that the presence of either of these honeys either prevents survival of break-through *S. aureus* colonies or prevents the attainment of mechanisms that enhance resistance to rifampicin.

## Discussion

Chronic wounds are an increasingly urgent health problem and bacterial infection plays a significant role in the inability of these wounds to heal [48]. Treatment of such infections often involves combinations of antibiotics in an effort to increase efficacy and stem antibiotic resistance. Honey has several antibacterial components and it is this property that is likely to explain why, unlike antibiotics, it does not induce resistance in bacteria. Here we show conclusively that the combination of clinically-approved manuka honey (Medihoney) and the antibiotic rifampicin has a synergistic effect on antibacterial activity against clinical isolates of *S. aureus,* including MRSA strains. We also show that MGO, a major antimicrobial compound in manuka honey [22], [23], is not solely responsible for the synergistic action. Moreover, while breakthrough colonies were obtained on plates containing rifampicin, the combination of rifampicin and Medihoney completely inhibited survival of *S. aureus*.

Recently, synergistic action between manuka honey and oxacillin was reported for *S. aureus* [44]; and between manuka honey and tetracycline, imipinem and mupirocin for *S. aureus* and *P. aeruginosa* [49]. These and our data support the idea of a combinational use of manuka honey and antibiotics for the effective treatment of chronic wound infections, particularly in cases where multidrug resistant organisms are present. Commercially-available honey dressings are also relatively inexpensive and non-toxic, which makes them attractive to use in combination with antibiotics.

A very recent study suggested a synergistic activity of rifampicin in combination with manuka honey. However, the high susceptibility of the MRSA strain used to rifampicin made it difficult to perceive increased susceptibility in the presence of honey [49]. Only one *S. aureus* strain, E-MRSA, was tested so we cannot rule out a strain specific issue in this case. Our study provides strong evidence that rifampicin in combination with maunka honey is synergistic across a range of *S. aureus* strains, including clinical isolates and MRSA.

As shown here and in previous studies, *S. aureus* can develop rifampicin resistance readily [29]. However, in the presence of sub-inhibitory concentrations of Medihoney or manuka honey, no rifampicin resistant *S. aureus* were detected. Whether honey acts to block the rifampicin resistance mechanism in *S. aureus* by preventing mutations in the gene encoding its target, the β subunit of RNA polymerase, or whether in the presence of both honey and rifampicin the bacteria do not survive long enough to develop resistance, remains unclear and needs further investigation. Regardless of the reason, our data here indicate that this combination treatment has potential in preventing the survival of *S. aureus* due to rifampicin resistance during therapy of skin infections and chronic wounds. In the longer term, this type of therapy may also reduce the rate of occurrence of rifampicin resistant bacteria in the clinic and the environment.

One of the predominant antibacterial compounds in manuka honey is methyglyoxal (MGO, [22], [23], [24]), which is formed by a non-enzymatic conversion of nectar-derived dihydroxyacetone [50]. However, the level of MGO present in honey appears to be considerably lower than that required to eliminate microbes treated with MGO alone [51]. Although a synergistic interaction between MGO and antibiotics against *Pseudomonas aeruginosa* has been reported [52], we did not find this with *S. aureus*. MGO in combination with rifampicin was only additive, not synergistic. Our results demonstrate that MGO is not solely responsible for the rifampicin-Medihoney synergistic activity. The botanical origin of honey influences its biological activity and many different antibacterial components have been identified in honey [53]. These components very likely interact with each other synergistically, additively or even antagonistically, so when isolated may have different effect on bacterial growth compared to their combined effect in honey. Interestingly, in the presence of sugar (equivalent to the sugar content of honey), the additive effect of MGO and rifampicin was significantly decreased compared to just MGO alone. This could be due to the growth-enhancing property of the sugar concentrations used here, reducing the antibacterial activity of MGO and rifampicin.

Various *in vitro* studies have shown synergistic effects between antibiotics and plant-derived pure compounds (such as baicalin, tellimagrandin I, epigallocatechin-gallate, or berberine; [41], [54], [55], [56]) or complex natural products (e.g. garlic extract; [57], [58]). However, unlike honey, none of these natural compounds or products has been successfully developed for clinical use as antibacterials. Importantly, concentrations of honey that have synergistic activity with rifampicin (6–8%) are easily achievable at the wound site, since typically honey dressings have honey concentrations of >80% [59], and are unlikely to decrease to such low concentrations even with large exudate volumes, as long as the dressings are changed at reasonable frequency.

Jenkins and colleagues reported that manuka honey caused a reversal of oxacillin resistance in MRSA. Treatment with 10% manuka honey led to a down regulation of *mecR1*, which codes for a two-component sensor/signal transducer protein that regulates the expression of *mecA* (encoding a penicillin-binding protein that mediates the oxacillin resistance, [44]). However, we could not detect a reversal of rifampicin resistance after treatment with Medihoney or manuka honey (**Fig. 3**). Rifampicin and oxacillin are members of different antibiotic classes and the resistance mechanisms are not related. Rifampicin resistance is typically due to a single-point mutation in the *rpoB* gene, resulting in an amino acid substitution in the rifampicin-binding site on RNA polymerase [34], [35], [36]. Thus, the potential of honey to reverse oxacillin resistance is likely related to the specific resistance mechanism against that antibiotic.

In conclusion, our results demonstrate a synergism between Medihoney and rifampicin against laboratory and clinical strains of *S. aureus* including MRSA strains. A combination of rifampicin and Medihoney maintained rifampicin susceptibility in *S. aureus,* which was rapidly lost in the presence of rifampicin alone. Our results support the potential of the combinational use of manuka honey and antibiotics in the treatment of *S. aureus*-related skin infections. The results of this study are encouraging, and controlled clinical studies are needed to define the efficacy of a Medihoney-rifampicin combination *in vivo*. Further study is also needed to determine the underlying mechanism of the synergistic action.

## Supporting Information

Table S1 (DOCX)
