# Supplementary material for: Synergism between Medihoney and Rifampicin against Methicillin-Resistant Staphylococcus aureus (MRSA)
Source: PLoS One. 2013 Feb 28;8(2):e57679. doi: 10.1371/journal.pone.0057679 (PMC3585195; doi:10.1371/journal.pone.0057679)
Supplement: Table S1 — (DOCX) [file pone.0057679.s001.docx]

**Table S1: Susceptibility of *S. aureus* to Medihoney, manuka and rifampicin**

|  | Minimum inhibitory concentration | | |
| --- | --- | --- | --- |
|  | Medihoney %(w/v) | manuka %(w/v) | rifampicin (µg/ml) |
| NCTC8325 | 8 | 8 | 0.039 |
| 04-229-2455 | 8 | 8 | 0.078 |
| 04-227-3567 | 8 | 8 | 0.039 |
| MW2 (USA400)^1^ | 8 | 8 | 0.039 |
| USA 300^1^ | 6 | 7 | 0.078 |
| IMVS67^1^ | 8 | 8 | 0.078 |
| RPAH18 (Aus-2)^2^ | 8 | 8 | 0.078 |

^1^community-aquired MRSA strains,  ^2^MRSA strains
